# Physalin A regulates the Nrf2 pathway through ERK and p38 for induction of detoxifying enzymes

**DOI:** 10.1186/s12906-019-2511-y

**Published:** 2019-05-09

**Authors:** Ji Min Shin, Kyung-Mi Lee, Hee Ju Lee, Ji Ho Yun, Chu Won Nho

**Affiliations:** 1 Natural Products Research Center, Korea Institute of Science and Technology (KIST) Gangneung Institute of Natural Products, Gangneung, Gangwon-do 25451 Republic of Korea; 2 Division of Bio-Medical Science &Technology, KIST School, Korea University of Science and Technology, Seoul, 02792 Republic of Korea; 3 Systems Biotechnology Research Center, Korea Institute of Science and Technology (KIST) Gangneung Institute of Natural Products, Gangneung, Gangwon-do 25451 Republic of Korea

**Keywords:** Physalin A, Cancer chemoprevention, Quinone reductase, NF-E2-related factor 2, Extracellular signal-regulated kinase, p38 mitogen-activated protein kinase

## Abstract

**Background:**

Physalin A isolated from *Physalis alkekengi var. franchetii* has been known to have many pharmacological properties. However, its effect through the Nrf2 pathway has not yet been elucidated. In the present study, we determined the effects of physalin A on cancer chemoprevention via the Nrf2 pathway.

**Methods:**

Experiments were performed in Hepa-1c1c7 and HepG2 cells. The quinone reductase (QR) activity assay was used to assess the activity of physalin A and other compounds isolated from *P. alkekengi*. The antioxidant response element (ARE) reporter assay was used to determine physalin A induced transcription of Nrf2 target genes, whereas the oligonucleotide pull-down assay was used to investigate Nrf2 binding to the AREs post physalin A treatment. Real-time PCR and western blotting were performed to determine the expression of Nrf2 target genes. Immunocytochemistry was used to determine Nrf2 localization after treatment with physalin A. Kinase inhibitors were used to test the involvement of Nrf2-targeting kinases and the role of ERK and p38 phosphorylation was confirmed using western blotting.

**Results:**

Physalin A significantly induced QR activity. As an upstream effector of QR, Nrf2 induced genes containing the ARE, which encode various antioxidants and detoxification enzymes. We observed that physalin A increased the expression of Nrf2 and its target genes in HepG2 cells. Moreover, we observed that physalin A-induced Nrf2 activation was regulated by ERK and p38 kinase in HepG2 cells.

**Conclusions:**

Taken together, we showed that physalin A increased detoxifying enzyme expression via activation of Nrf2 and its target genes. These results imply that physalin A could be a potential chemopreventive agent for liver diseases, as well as cancer.

## Background

Liver cancer, the fifth-most common cancer worldwide, is the third-most common cause of mortality due to cancer [1]. Furthermore, liver cancer-associated mortality in both men and women is higher than the diagnostic rates [2]. Thus, prevention of liver cancer is of utmost importance. Synthetic or natural pharmaceutical agents mainly used, especially, traditional medicine is useful to prevent the development of various diseases, including cancer due to their safety and affordability [3–5].

Phytochemicals, especially plant-derived compounds, are used in clinical trials as cancer chemopreventive agents [6]. For example, sulforaphane is a representative phytochemical obtained by hydrolysis of glucoraphanin, which is abundant in broccoli. Sulforaphane inhibits cancer growth and the overall carcinogenetic process by inducing phase II enzymes, including quinone reductase (QR, NAD(P)H: quinone oxidoreductase) and glutathione S-transferases (GSTs) [6, 7].

**Fig. 1:**
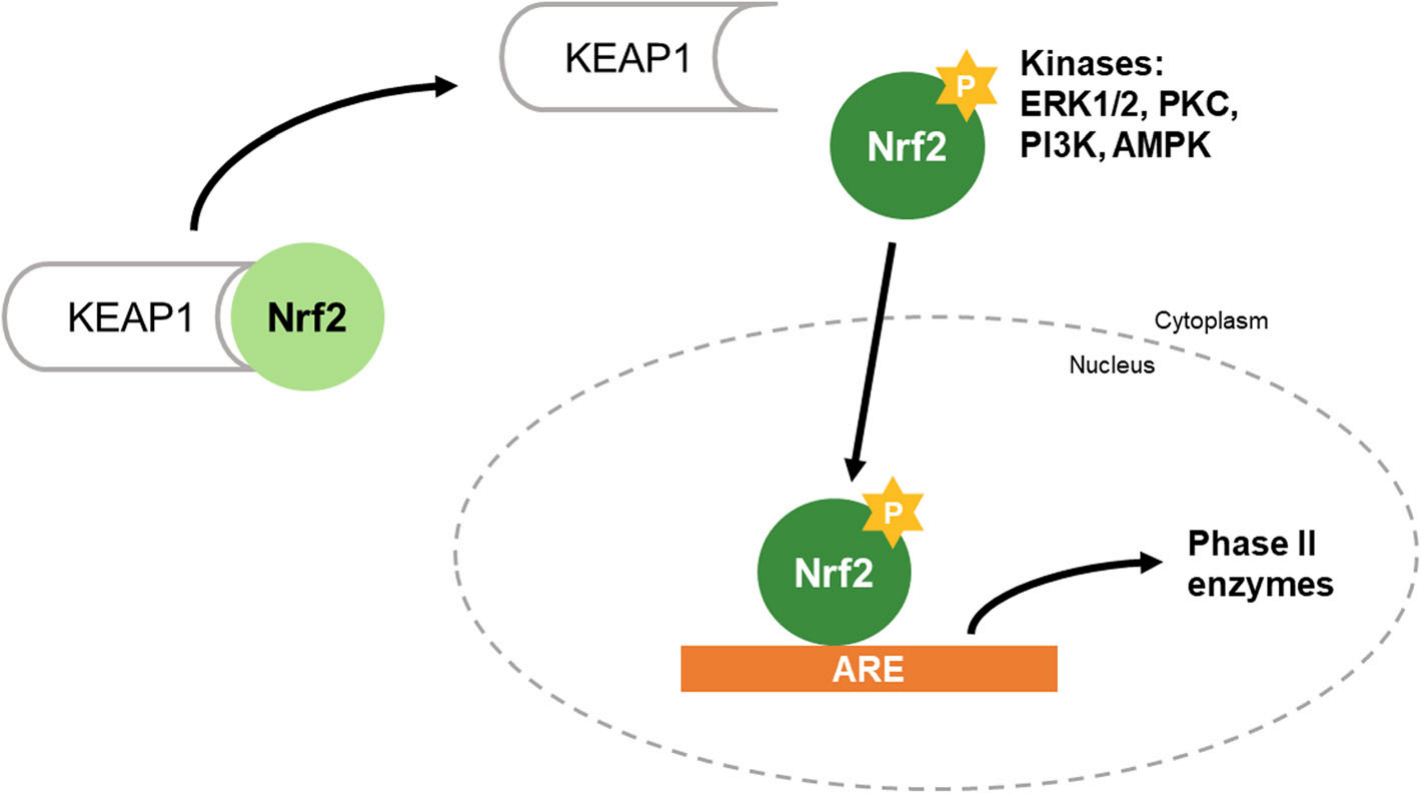
Mechanism of the Nrf2 pathway. Under oxidative stress condition, Nrf2 separates from Keap1 via phosphorylation of it by various kinases. Nrf2 translocated into nucleus and binds to ARE region on promoter of the target gene including HO-1 and NQO-1

Nuclear factor erythroid 2-related factor 2 (Nrf2), a member of the basic leucine zipper transcription factor family, regulates the expression of genes containing the antioxidant response element (ARE) in the promoter region, which are related to antioxidation and detoxification. Under normal conditions, Nrf2 dimerizes with Kelch-like ECH-associated protein 1 (Keap1) [8] (Fig. 1). Nrf2 protects cells from stress inducers such as endogenous reactive molecules, radiation, and environmental toxins [9]. When cells are exposed to stress, Nrf2 separates from Keap1 in the nucleus and activates its target genes [10]. Several kinases such as ERK1/2, PKC, PI3K, and AMPK regulate Nrf2 expression [11–14].

Nrf2 targets such as heme oxygenase-1 (HO-1), NAD(P)H quinone oxidoreductase (NQO-1), and GSTs encode phase II detoxification enzymes [15]. HO-1, a cytoprotective enzyme, regulates antioxidative and inflammatory responses, and NQO-1 is involved in detoxification [8], which may inhibit cancer initiation by detoxifying and eliminating carcinogens [16]. In addition, previous studies have reported an association of the QR-encoding gene with risks of developing various cancers [17, 18]. Therefore, QR can be targeted for developing potential cancer chemopreventive agents.

*Physalis alkekengi* var. *franchetii (Solanaceae)* is found in East Asia and is known to ameliorate otitis media, fever, sore throat and renal diseases [19] [20]. Physalin A, one of the major bioactive compounds isolated from *P. alkekengi* possesses many pharmacological properties, including antifungal, anti-cough, anti-inflammatory, and analgesic activities in vivo *and* in vitro [21]. Physalin A induces apoptotic cell death in various cell lines and induces G2/M cell cycle arrest in human lung cancer cells [22, 23]. However, the chemopreventive effect of physalin A via the Nrf2 pathway has not yet been elucidated.

In this study, we investigated the effect of *P. alkekengi* and physalin A on cancer chemoprevention via the Nrf2 pathway. Physalin A induced Nrf2 and its target genes encoding HO-1 and NQO1 via ERK and p38 kinases in HepG2 cells.

## Methods

### Chemicals and reagents

*P. alkekengi* was purchased from a Kyungdong oriental herbal market, Seoul, Republic of Korea. The voucher specimens (ND4) have been deposited at the Systems Biotechnology Research Center, KIST, Gangneung Institute of Natural Products, Republic of Korea. This plant identified by Dr. Hak Cheol Kwon who responsible for KIST natural products library at KIST Gangneung, institute of natural products. Dried *P. alkekengi* (2.5 kg) were extracted using 95% ethanol for 4 h by reflux. After filtration, the ethanol were evaporated in a vacuum to obtain the ethanol extract (203 g), which was suspended in distilled water and partitioned using n-hexane, ethyl acetate, and n-butanol. The ethyl acetate fraction (15 g) was chromatographed on a Sephadex LH-20 column, eluted using methanol to obtain five fractions (fractions 1–5). Physalin A was re-chromatographed from fraction 3 using Sephadex LH-20 (methanol) and RP-18 gel [methanol-water (40 → 70%, *v*/v)] column chromatography to obtain white crystals. The chemical structures of compound 1 was determined by ^1^H and ^13^C nuclear magnetic resonance and the results were compared with published data [24].

Dulbecco’s high glucose modified Eagle medium (DMEM) (Hyclone, Logan, UT, USA), fetal bovine serum (FBS), 100 U/ml penicillin, and 100 μg/ml streptomycin were obtained from Thermo Scientific (Waltham, MA, USA). 3-(4,5-Dimethyl-2-thiazolyl)-2,5-diphenyl-2H-tetrazolium bromide (MTT) and bovine serum albumin were purchased from Sigma-Aldrich (St, Louis, MO, USA).

### Cell culture

Hepa-1c1c7 (mouse hepatoma cells) and HepG2 (human hepatocellular carcinoma cells) were purchased from the American Type Culture Collection (ATCC, Manassas, VA, USA). The cells were maintained at sub-confluence in the presence of 95% air and 5% CO_2_ in a humidified atmosphere at 37 °C. DMEM and α-MEM were used for HepG2 and Hepa-1c1c7 cell cultivation, respectively. The media were supplemented with 10% FBS, 100 U/ml penicillin, and 100 μg/ml streptomycin.

### Quinone reductase (QR) assay

**Fig. 2:**
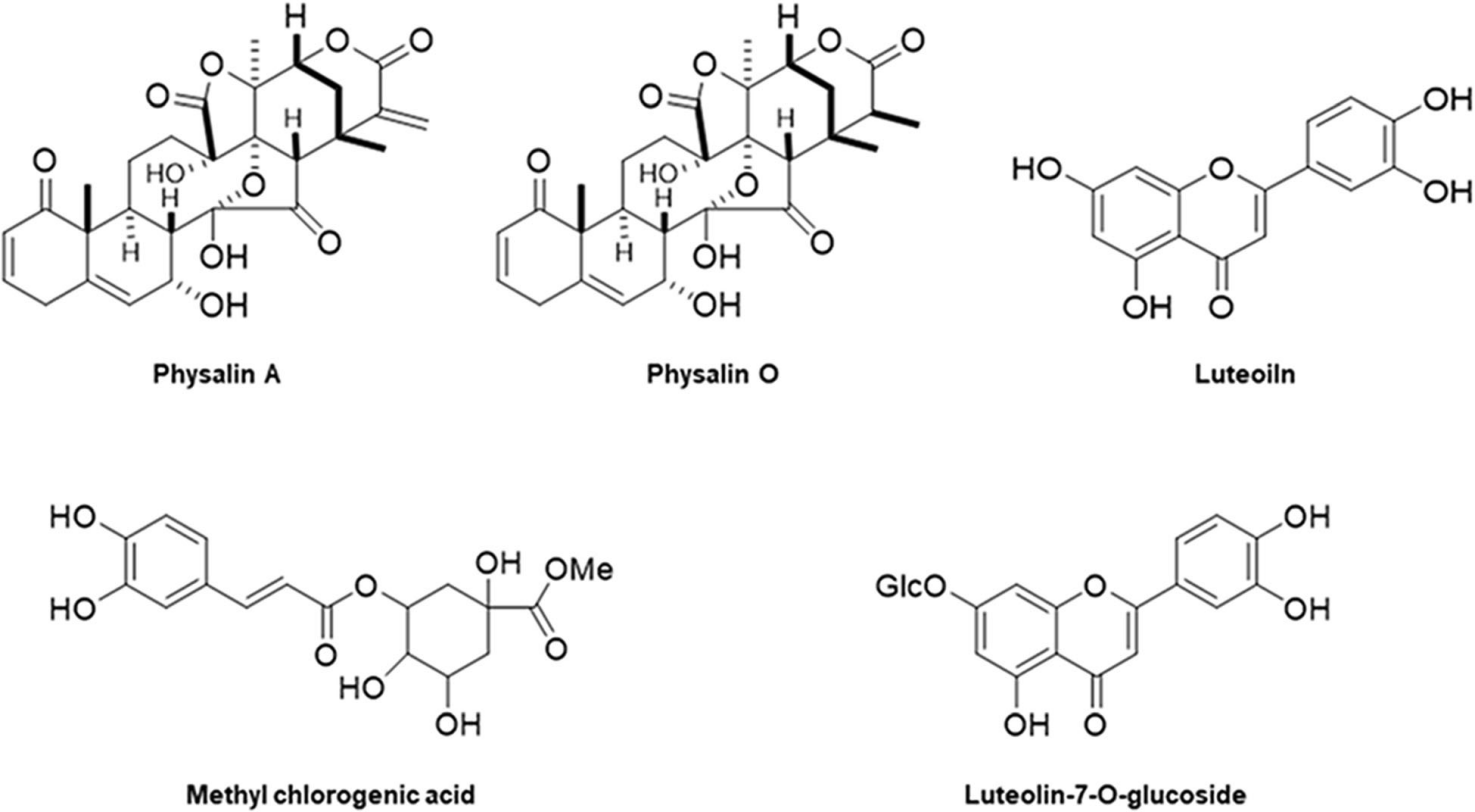
Structure of physalin A, physalin O, luteolin, methyl chlorogenic acid, and luteolin-7-O-glucoside isolated from *P. alkekengi*

Specific QR activity was measured using a previously reported QR assay [25, 26], after brief modifications. Hepa-1c1c7 cells (1 × 10^4^ cells/well) were plated on 96-well culture plates and incubated for 24 h before treatment with five compounds isolated from *P. alkekengi* including physalin A (Fig. 2). The absorbance at 610 nm was determined five times at 50 s intervals using a Synergy HT multi-microplate reader (Bio-Tek Instruments, Winooski, VT, USA).

### Cell viability assay

Cell viability was measured using the WST-1 cell proliferation assay kit (EZ-Cytox; Daeil Lab, Seoul). HepG2 cells (1 × 10^4^ cells/well) were plated in 96-well culture plates and incubated at 37 °C for 24 h, followed by treatment with various concentrations of physalin A. After 24 h of treatment, we added 1/10 diluted EZ-Cytox solution to each well and incubated for 1 h 20 min. Then, absorbance was measured at 450 nm using Synergy HT multi-detection microplate reader.

### Preparation of nuclear and cytosolic fractions

HepG2 cells (6 × 10^5^ cells/well) were plated in 6-well culture plates and incubated for 24 h prior to physalin A treatment. Nuclear and cytosolic fractions were prepared using a nuclear extraction kit (Cayman, Ann Arbor, MI, USA).

### Western blot analysis

HepG2 cells (6 × 10^5^ cells/well) were plated in 6-well culture plates and incubated for 24 h prior to physalin A treatment. Cell lysate was prepared using ice-cold radioimmunoprecipitation assay (RIPA) buffer (Thermo Scientific) containing phenylmethane sulfonyl fluoride (PMSF) and a protease inhibitor cocktail (Sigma, St. Louis, MO, USA), followed by centrifugation for 25 min at 4 °C. Protein concentrations were determined using protein assay dye reagent concentrates (Bio-Rad, Hercules, CA, USA). Total cell lysates, and nuclear and cytosolic fractions were loaded on 10% sodium dodecyl sulfate polyacrylamide gels, electrophoresed, and then transferred to polyvinylidene difluoride membranes (Bio-Rad). The membranes were blocked using 3% bovine serum albumin (BSA), followed by incubation with primary antibodies against the following proteins: *β*-actin, NQO-1, HO-1, LaminB (Santacruz Biotechnology, Santa Cruz, CA, USA); Nrf2 (Abcam, Cambridge, MA, USA); ERK, p-ERK, p38, and p-p38 (Cell Signaling Technology, Denvers, MA, USA) in 3% BSA. The western blots were developed using SuperSignal™ West Femto maximum sensitivity substrate reagent (Thermo Scientific).

### Total RNA extraction and quantitative reverse transcription-polymerase chain reaction (qRT-PCR)

Total RNA was extracted from HepG2 cells using the RNeasy mini kit (Qiagen, Hilde, Germany) according to the manufacturer’s instruction. cDNA was synthesized using the PrimeScript™ first strand cDNA synthesis kit (Takara, Shiga, Japan) according to the manufacturer’s instruction. qRT-PCR analysis was performed using the LC480 detection system (Roche, Basel, Switzerland).

### ARE reporter assay

HepG2 cells (1 × 10^5^ cells/well) were plated in 24-well culture plates and incubated for 24 h, followed by physalin A treatment. The cells in each well were transfected with 0.4 μg ARE-luc reporter construct containing human NQO-1 sequences and 50 ng of pRL-CMV transfection control vector using the TransIT-2020 transfection reagent (Mirus Bio, Madison, WI, USA) according to the manufacturer’s instructions. The reporter assay was performed using a dual luciferase assay kit (Promega, Madison, WI, USA) and Synergy HT multi-detection microplate reader.

### Oligonucleotide pull-down assay

HepG2 cells (3 × 10^6^ cells/well) were plated in 100-mm culture dishes and incubated for 24 h, followed by physalin A treatment. The cells were lysed in HKMG buffer (10 mM HEPES (pH 7.9), 100 mM KCl, 5 mM MgCl_2,_ 1 mM dithiothreitol (DTT), 10% glycerol and 0.5% NP-40), and the cell lysates were incubated overnight with biotinylated oligonucleotides. The sequence of the oligonucleotide is as follows: 5′-AAATCGCAGTCAC- -AGTGACTCAGCAGAATCTGAGCCTAGG-3′. To obtain the nucleotide bound protein, the samples were incubated with streptavidin-agarose resin (Thermo Scientific) for 6 h at 4 °C, and then washed with HKMG buffer. The washed samples were used for western blot analysis.

### Immunocytochemistry

The cellular distribution of Nrf2 was determined using immunofluorescence assay and confocal microscopy as previously described [27]. In brief, HepG2 cells (2 × 10^3^ cells/well) were plated on glass coverslips in 24-well plates, and incubated for 24 h prior to treatment. Next, the cells were incubated with primary antibody against Nrf2 (1:150; Cambridge, UK) overnight at 4 °C. Alexa Fluor 488-conjugated anti-rabbit (Invitrogen, Carlsbad, CA, USA) secondary antibody was used at 1:200 dilution for 1 h at room temperature. Antibodies were diluted in phosphate buffered saline-Tween 20 (PBST) containing 5% normal serum and 0.3% Triton X-100. Images were obtained using a Leica TCS SP5 confocal system (Leica, Wetzlar, Germany).

### Statistical analysis

Results were presented as mean ± standard error of the mean (SEM). Statistical significance was determined using one-way analysis of variance (ANOVA) and Dunnett’s multiple comparison test. *P* < 0.05 (calculated using GraphPad Prism version 7.00) indicated statistically significant difference with respect to the control group.

## Results

### Physalin A induces specific QR activity

**Fig. 3:**
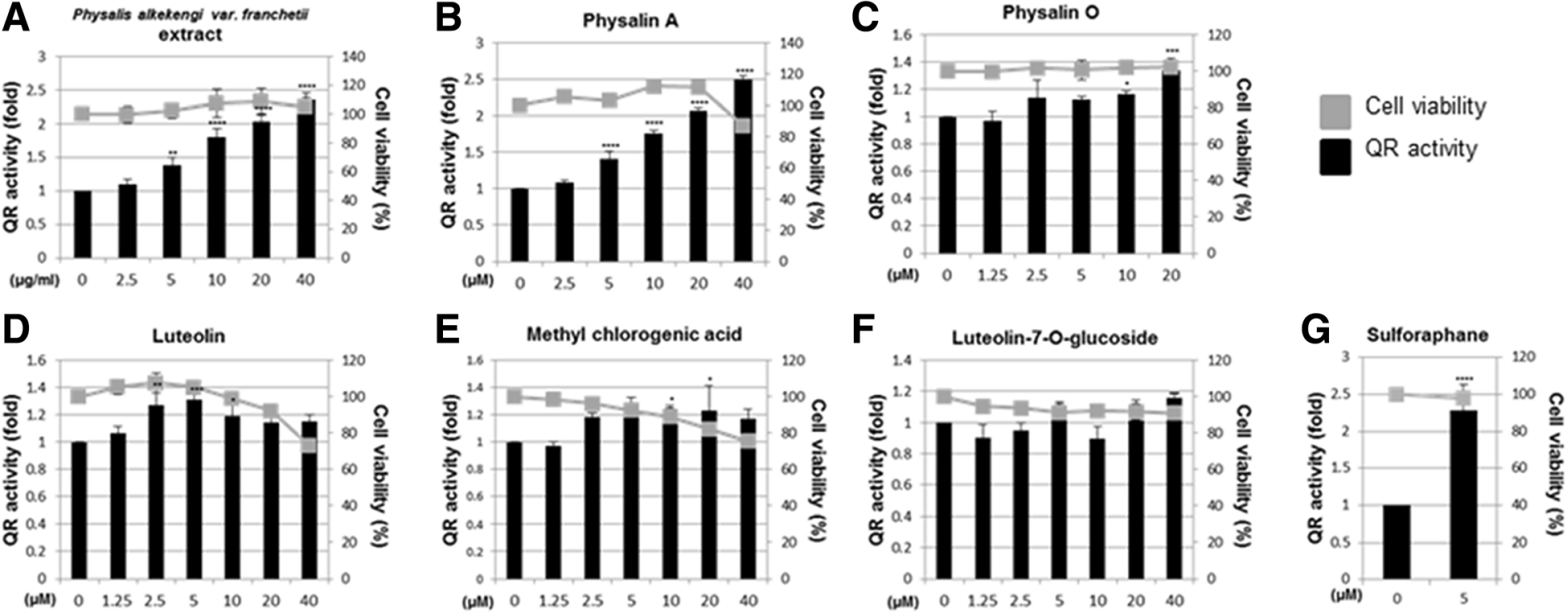
Induction of QR-specific enzymatic activity in Hepa-1c1c7 cell line (**a**). QR assay and viability assay of Hepa1c1c7 cells treated with *P. alkekengi* (**b**) physalin A, (**c**) physalin O, (**d**) luteolin, (**e**) methyl chlorogenic acid, and (**f**) luteolin-7-O-glucoside. (**g**) QR assay and viability assay of sulforaphane-treated Hepa-1c1c7 cells. The cells treated for 24 h with 5 μM sulforaphane as a positive control. (*: *p* < 0.05, **: *p* < 0.01, ***: *p* < 0.001, ****: *p* < 0.0001)

QR activity is a measure of the chemopreventive effect of any compound [7, 28]. We first assessed the specific QR activity of the *P. alkekengi* extract and five compounds derived from this plant in Hepa-1c1c cells. Results showed that the extract and only physalin A increased specific QR activity in a dose-dependent manner (Fig. 3a-b). Other compounds, such as physalin O, luteolin, methyl chlorogenic acid, and luteolin-7-O-glucoside did not significantly increase QR activity (Fig. 3c-f). The *P. alkekengi* extract and the isolated compounds did not significantly affect cell viability. Sulforaphane was used as a positive control in these experiments. These results showed that physalin A is an active component responsible for induction of QR activity.

### Physalin A induces NQO1 transcription in HepG2 cells

**Fig. 4:**
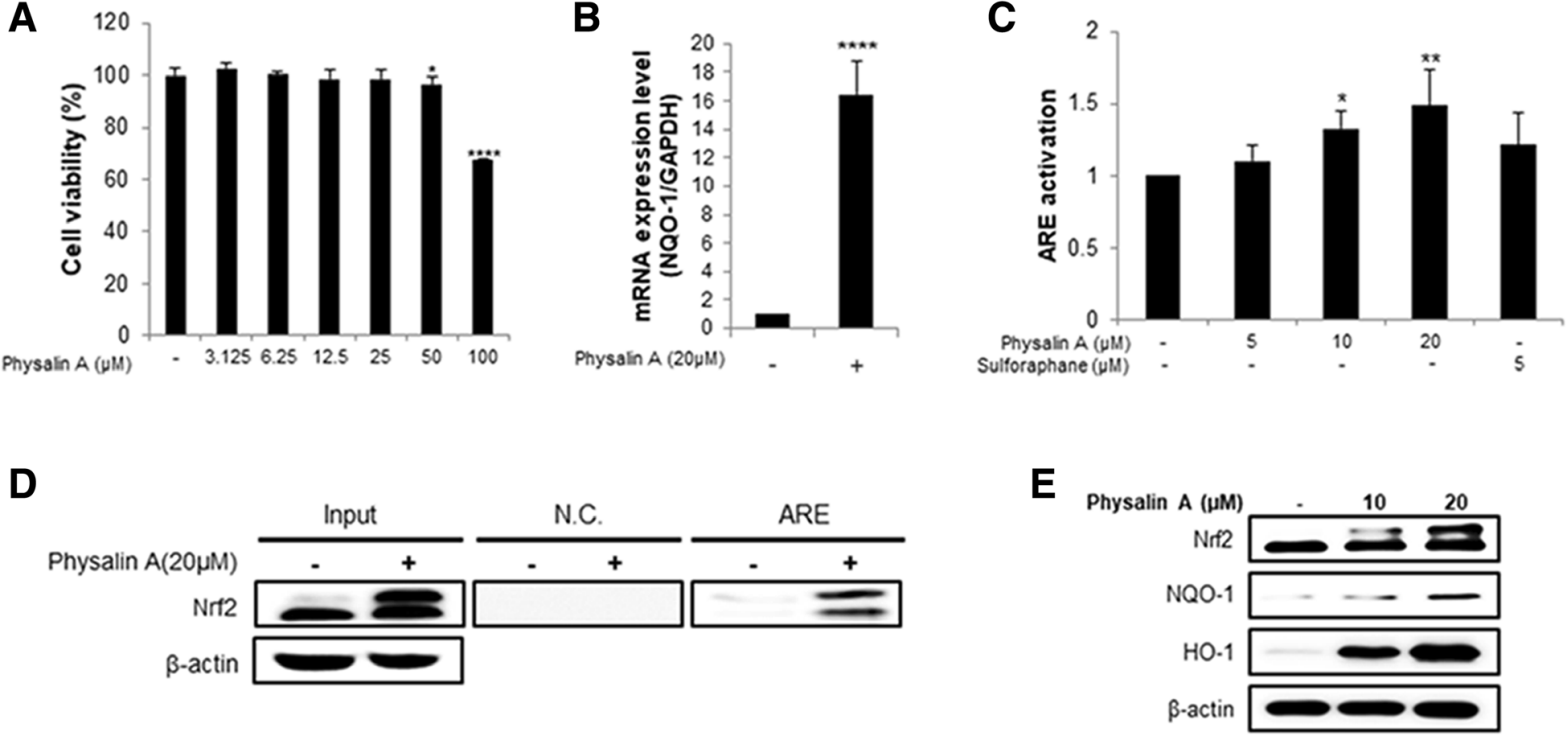
Physalin A induces NQO-1 transcription in HepG2 cells. **a** Viability of physalin A-treated HepG2 cell line. Cells treated with various concentration physalin A for 24 h. **b** NQO-1 expression was measured using real-time PCR. **c** ARE transcriptional activity of physalin A-treated HepG2 cells. The cells were treated with 5, 10, 20 μM physalin A for 24 h and cell lysates were used for luciferase assay. **d** Oligonucleotide pull-down assay in HepG2 cells with ARE element. The cells were treated 20 μM physalin A for 4 h and then harvested to determine ARE-binding activity. **e** Western blot analysis to assess the expression of Nrf2 and its target genes, NQO-1 and HO-1 in HepG2 cells. The cells were treated with 10 and 20 μM physalin A (*: *p* < 0.05, **: *p* < 0.01, ***: *p* < 0.001, ****: *p* < 0.0001)

Since physalin A was the active component required for QR activity, we performed cell viability assay using 3.125–100 μM physalin A (Fig. 4a) to determine the non-cytotoxic concentration range that can be used in further experiments involving HepG2 cells. No significant cytotoxicity was observed below 25 μM (Fig. 4a).

To investigate whether physalin A induces QR activity through the Nrf2 pathway, we measured Nrf2 mRNA level and ARE activation. Nrf2 upregulated genes encoding phase II detoxification enzymes, such as NQO-1, by binding to the ARE in the target gene’s promoter regions [8]. qRT-PCR and western blotting showed that physalin A increased NQO-1 mRNA expression (Fig. 4b) and the protein levels of Nrf2 target genes, respectively (Fig. 4e). To determine whether physalin A activated the ARE by promoting Nrf2 binding, we performed an oligonucleotide pull-down assay and a reporter assay using a construct containing the ARE (Fig. 4d). Also, we observed that the binding of Nrf2 to ARE was increased by physalin A in HepG2 cells in a dose-dependent manner (Fig. 4c).

### Physalin A induces Nrf2 expression and nuclear accumulation

**Fig. 5:**
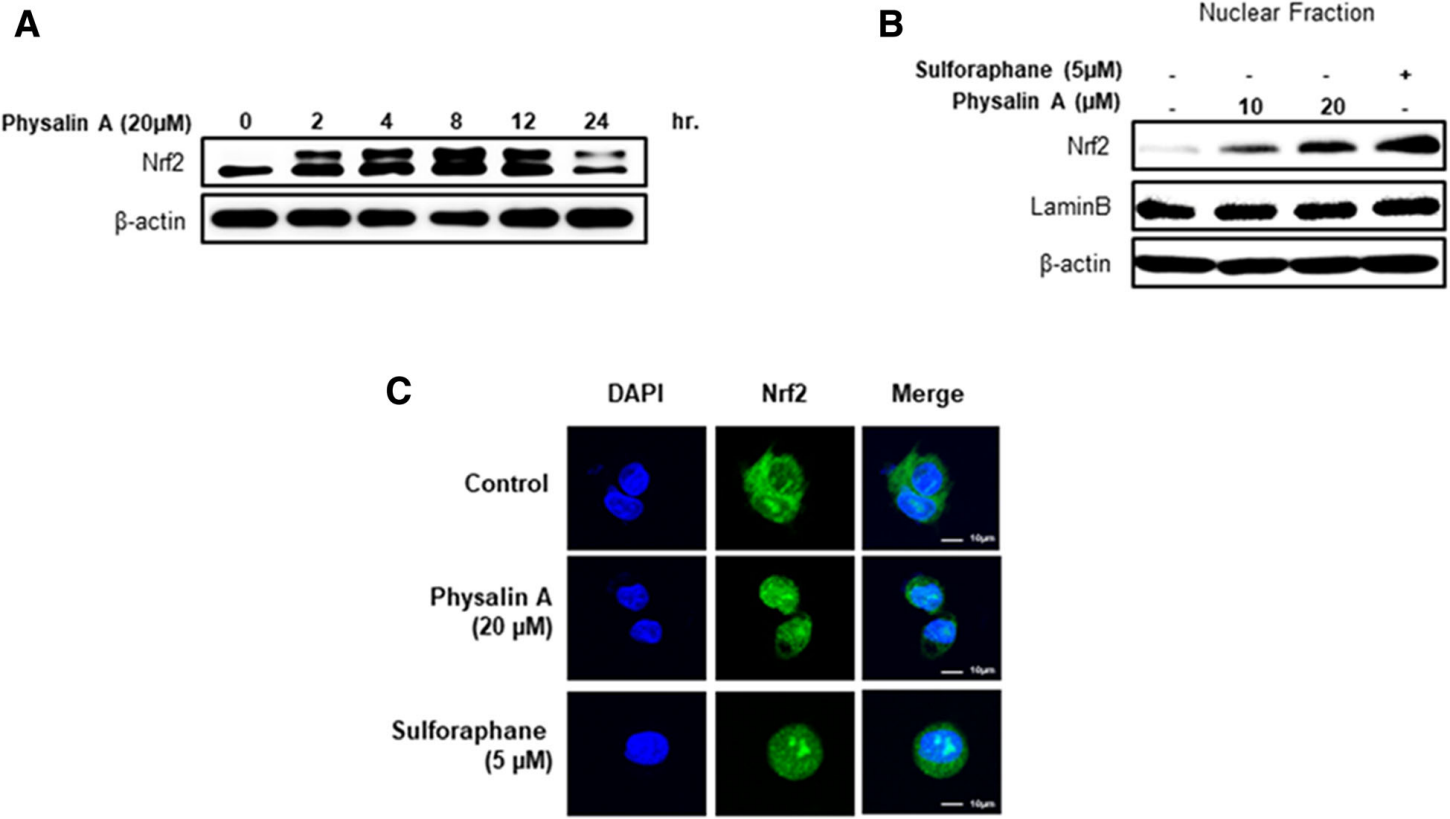
Physalin A induces Nrf2 nuclear expression and activation. **a** Western blot analysis of Nrf2 expression in HepG2 cells. The cells were treated with 20 μM physalin A for 0, 2, 4, 8, 12, 24 h. **b** Western blot analysis of Nrf2 expression in the nuclear fraction. The cells were treated with 10 and 20 μM physalin A for 4 h. **c** Immunocytochemistry showing Nrf2 nuclear accumulation in HepG2 cells. The cells were treated with 20 μM physalin A for 4 h

To confirm that physalin A increases Nrf2 expression and nuclear accumulation, we assessed Nrf2 expression at various time points and monitored nuclear translocation of Nrf2 from the cytosol using fluorescent dyes. Nrf2 expression in a time-dependent manner (Fig. 5a) when the cells were treated with 20 μM physalin A. We also observed that physalin A increased Nrf2 nuclear accumulation in dose-dependent manner (Fig. 5b-c) when the cells were treated for 4 h. Thus, these results showed that physalin A induces Nrf2 expression and nuclear accumulation.

### Physalin A induces Nrf2 activation via ERK and the p38 kinase

**Fig. 6:**
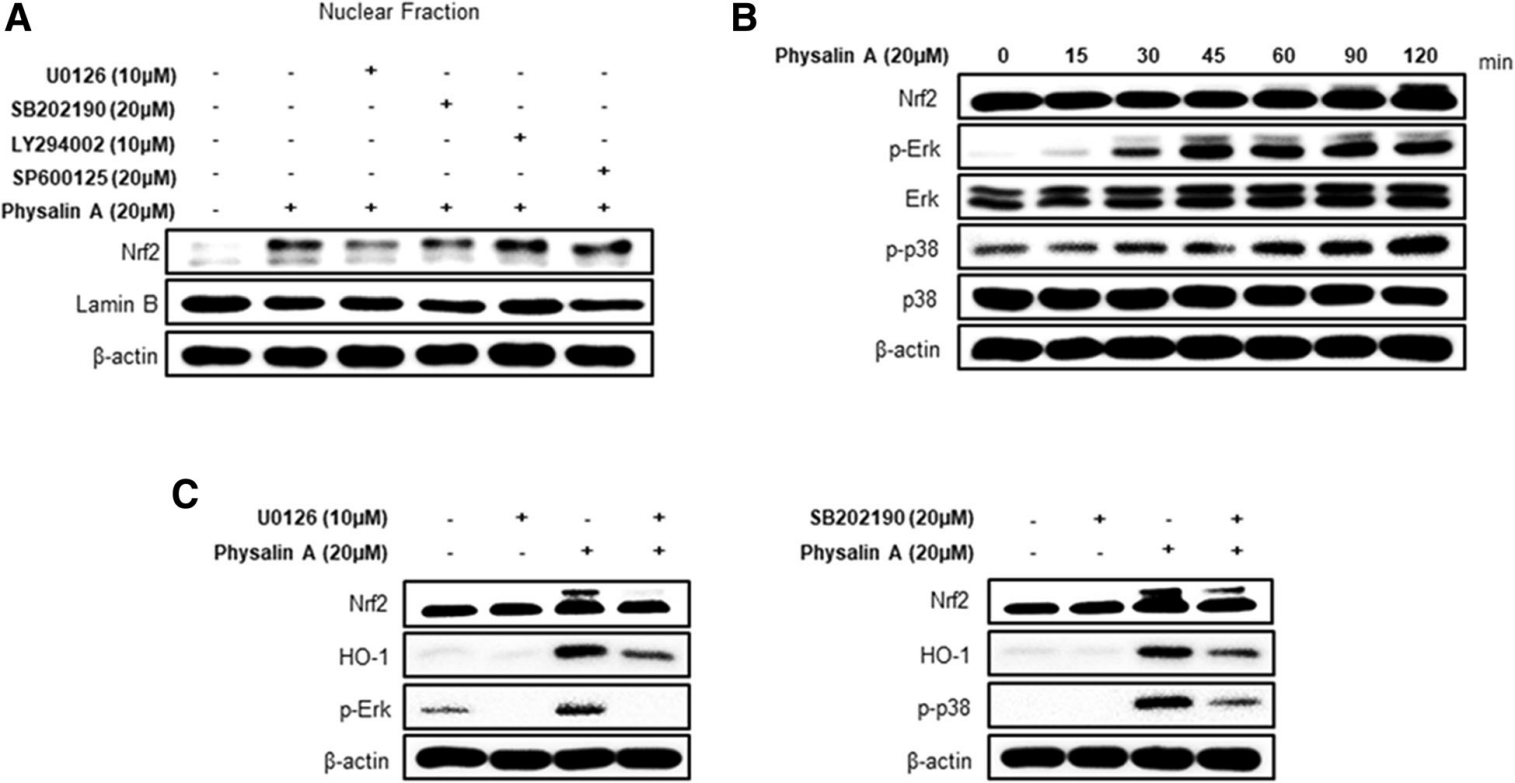
Physalin A induces Nrf2 and target gene expression through ERK and p38 kinase activation. **a** Western blot analysis of Nrf2 expression in HepG2 cells pretreated with U0126 (MEK inhibitor), SB202190 (p38 inhibitor), LY294002 (PI3K inhibitor), and SP600125 (JNK inhibitor) for 1 h, followed by treatment with 20 μM physalin A for 4 h. **b** Western blot analysis of phospho-ERK and phospho-p38 expression in HepG2 cells treated with 20 μM physalin A for 0, 15, 30, 45, 60, 90, and 120 min. **c** Western blot analysis of Nrf2 target gene expression in HepG2 cells pretreated with U0126 and SB202190 for 1 h and then with 20 μM physalin A for 8 h

A previous study showed that multiple kinases are involved in the translocation of Nrf2 to the nucleus [11–14]. To identify the kinases required for physalin A-mediated Nrf2 translocation, we assessed Nrf2 expression after pre-treatment with several following kinase inhibitors: LY294002 (inhibitor of PI3K), SP600125 (inhibitor of JNK), U0126 (inhibitor of ERK), or SB202190 (inhibitor of p38). Particularly, treatments with U0126 (inhibitor of ERK) and SB202190 (inhibitor of p38) reduced Nrf2 nuclear accumulation (Fig. 6a). To further confirm if ERK and p38 regulate Nrf2 activation, the phosphorylated-forms of ERK and p38 were investigated in a time-dependent manner. We observed that physalin A increases ERK and p38 phosphorylation. We also determined the expression of HO-1, one of the target genes, in cells treated with physalin A and U0126 or SB202190. Results showed that the expression of Nrf2 and its target genes was considerably reduced after co-treatment with the inhibitor compared to treatment with physalin A alone (Fig. 6c). Thus, these results suggest that physalin A activates the ERK and p38 kinases to induce Nrf2 nuclear translocation.

## Discussion

Physalin A, isolated from *P. alkekengi*, is a triterpenoid with a steroid skeleton, which shows anti-cancer activity in various cancer cell lines [21, 23, 29, 30]. Terpenoids are organic chemicals composed of isoprene units and many of them are of plant origin [31]. The majority of terpenoids also possess pharmacological properties, including anti-cancer, anti-inflammatory, anti-viral, and anti-fungal activities [31, 32]. Several studies have shown that terpenoids show anti-cancer activity via MAPK phosphorylation, including ERK and p38, in various cancers [33–35]. In this study, physalin A increased the expression of detoxification enzymes by activating the Nrf2 pathway via ERK and p38. Therefore, we speculated that these results might be related to terpenoid structure. Interestingly, although physalin A and O have a similar structure, physalin A activated Nrf2 expression and induced phase II detoxification enzymes, whereas physalin O showed no significant effect on QR activity. The sole difference in structure between the two compounds is a double bond between C_25_ and C_27_ [24], which possibly contributed to the difference in biological activity.

We observed that physalin A activated the Nrf2-ARE signaling pathway through ERK and p38 phosphorylation at different time points (Fig. 6b). While ERK phosphorylation was observed from 15 min and peaked at 45 min, p38 phosphorylation increased from 60 min onwards. These results suggest that ERK phosphorylation triggers Nrf2 translocation at early time points (within 30 min), followed by p38 kinase phosphorylation at later time points, which induces Nrf2 translocation and nuclear accumulation. Furthermore, our results suggest that it is not possible to exclude the effect of other transcription factors and upstream signaling molecules in modulating the expression of Nrf2 and its target genes, because ERK and p38 kinase inhibitors do not completely abrogate physalin A-induced Nrf2 expression and nuclear accumulation. Further studies on the differential temporal regulation of these kinases and involvement of other factors are required.

As an upstream key factor that increases detoxifying enzyme activity, Nrf2 regulates the expression of several target genes. Here, we showed that physalin A induces Nrf2 activation and the expression of its target genes, NQO-1 and HO-1. However, these target genes were not expressed to similar extents at the same time point. Previously, [36] demonstrated differential regulation of HO-1 and NQO-1 by ethanol. Ethanol-induced HO-1 expression was regulated by Nrf2, HIF-1α, and JNK, whereas that of NQO-1 was regulated by Nrf2 and Src kinase. Thus, we speculated that the target genes of Nrf2 induced by physalin A, including NQO-1 and HO-1, may be regulated by different kinases and other factors, respectively.

Post nuclear translocation, Nrf2 may be phosphorylated by an upstream kinase or stabilized by Keap1 modifications. Several dietary phytochemicals cause Keap1-specific cysteine thiol group oxidation or chemical modification. Sulforaphane induces phosphorylation of Nrf2 upstream of the p38 MAP kinase, and Nrf2 stabilization and nuclear accumulation by specific modifications of Keap1 [12, 37, 38]. However, we have not investigated whether physalin A regulates conformational changes of Keap1 in the cytosol. The additional study of the keap1 modification should be required in the future studies.

## Conclusions

**Fig. 7:**
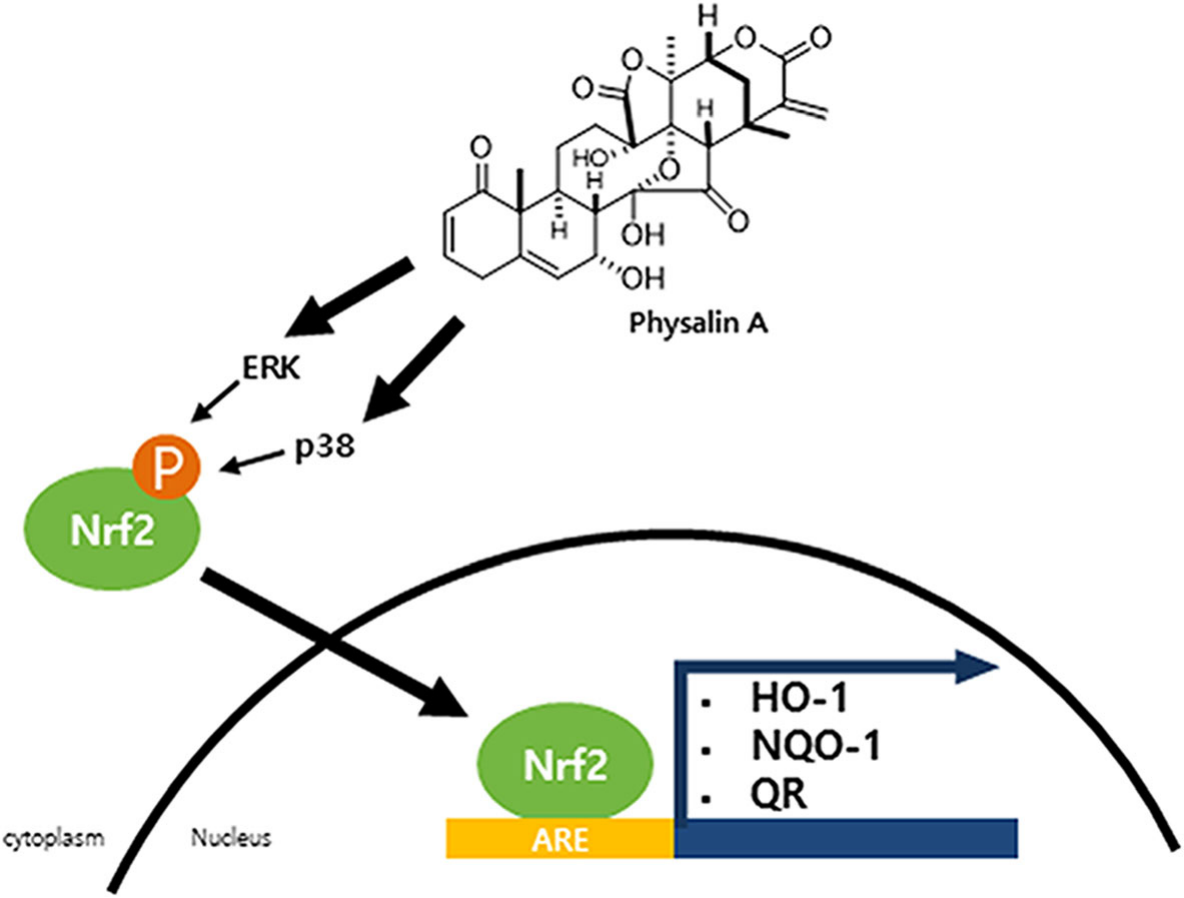
The hypothetical Nrf2 pathway model induced by physalin A. Physalin A induces the Nrf2 activation and nuclear translocation through Erk or p38 kinase phosphorylation. In the nucleus, ARE binding of Nrf2 induces detoxification gene expression such as NQO-1, HO-1, and QR.

In conclusion, we showed that physalin A increased detoxifying enzyme expression via activation of Nrf2 and its target genes. The results of the QR assay showed that physalin A might suppress cancer development at the initial stage of carcinogenesis by regulating the activity of phase II detoxification enzymes (Fig. 7). These results showed that physalin A is a potential chemopreventive agent that regulates Nrf2 pathway in liver cancer.

## Abbreviations

ARE: Antioxidant response element
p38: p38 mitogen-activated protein kinase
ERK1/2: Extracellular signal-regulated kinase
JNK: C-Jun N-terminal kinase
PKC: Protein kinase C
AMPK: AMP-activated protein kinase
PI3K: Phosphatidylinositide 3-kinases
HO-1: Heme oxygenase 1
Nrf2: Nuclear factor erythroid 2-related factor 2
NQO1: NAD(P)H quinone dehydrogenase 1
QR: Quinone reductase
